# Tax and Globalisation: Toward a New Social Contract

**DOI:** 10.1093/ojls/gqae010

**Published:** 2024-03-30

**Authors:** Tsilly Dagan

**Affiliations:** Professor of Taxation Law, University of Oxford, Faculty of Law; Fellow, Worcester College; and Raoul Wallenberg Professor of Law, Bar Ilan University, Faculty of Law

**Keywords:** Taxation, Globalisation, Mobility

## Abstract

Taxation and representation are famously linked in the coercive co-authored project of political governance described through the social contract metaphor. Globalisation transforms this canonical account of the state. Many people can relocate and operate beyond state borders, consuming goods and services publicly offered by other jurisdictions. Expanding people’s opportunities to satisfy their preferences and pursue their goals supports their liberty. Yet, it also limits the ability of states to collect taxes so as to provide necessary public goods and secure justice, jeopardising the bond between taxation and equal membership in a political community. The challenge for taxation under globalisation is to revitalise the very basis of the social contract. Ideally, the new social contract should support the states’ just institutions and the collective self-determination of their members without rolling back the opportunities people have acquired through globalisation.

## 1. Introduction

The state is the means through which people can pursue their collective self-determination. Taxation plays a major role in this project as the key instrument states use to finance their operations and ensure the overall justice of the system. By anchoring the fiscal elements, taxation allows states to use their coercive power to promote the collective will of their constituents. This function clarifies why taxation and representation are famously linked in the coercive co-authored project of political governance denoted by the social contract metaphor.^1^

The global perspective markedly affects this tie between national tax systems and political governance. While globalisation entails more opportunities for people, it also presents them as well as their states with mounting challenges. The mobility of taxpayers and resources, the infamous overlaps, gaps and frictions between national tax systems, and the resulting inter-jurisdictional competition and tax avoidance have long been recognised as obstacles to the states’ exclusive coercive power to tax, raising questions such as double taxation, no taxation, tax enforcement and the allocation of taxing rights among countries. My central question in this article is: what happens to the social contract idea when people operate in more than one jurisdiction, easily able to relocate and/or benefit from publicly provided goods and services simultaneously provided by multiple states and de facto picking and choosing among them to construct their own customised bundles of goods and services?

Following globalisation, many people operate beyond state borders, with their lives and activities spreading beyond a single state. People can not only relocate, but also reside in one jurisdiction while taking advantage of regulatory regimes or other goods and services publicly offered in others. They can thus unbundle the packages of goods and services offered by states and consume them *à la carte*—reside in one state but study, invest, trade, litigate and so forth in many others.

What facilitates this diversification is that, under globalisation, states simultaneously interact not only with their own constituents, but also with non-members. States can invite outsiders to gain full membership as citizens but can also limit their interaction with outsiders to the provision of only some of their goods and services for a fee (as in the case of incorporation fees) or provide some of their goods and services in exchange for resources domestically in demand, such as capital (in the case of foreign direct investment, FDI), talent (in the case of athletes or scientists) or simply positive spillovers (as in the case of research and development). As country clubs can sell day passes for a fee, states can (and do) invite foreigners to visit, rent, invest, establish businesses in the country, trade with locals, work, study or even get life-saving medical treatment without becoming full ‘members’.

Globalisation thus fundamentally challenges the idea of a bond between states and their constituents through the social contract. Ideally, under the social contract metaphor the state uses the coercive power entrusted to it to provide specific goods and services and justly (re)distribute resources while treating its constituents with equal concern and respect. In open economies, however, when barriers between states are removed, the state’s coercive power is at risk of unravelling, not only because of its declining capability to impose and enforce taxes, but also due to its fading authority. Inter-jurisdictional competition, alongside the ability to unbundle what the state offers, undercuts equal concern and respect and distributive justice, thus undermining the states’ legitimate use of their coercive power to tax. Though seemingly merely an expansion of people’s choices, unbundling risks severing the bonds between taxation and equal membership in a political community. Potentially, then, unbundling undermines people’s co-authorship in their collective self-determination.

The two features of the global regime at the core of this process are mobility and fragmentation. Mobility allows people to relocate and thus choose between competing jurisdictions *en bloc*. Fragmentation goes further by allowing people to engage with multiple jurisdictions simultaneously, mixing and matching the public goods and services they consume across jurisdictions. Both mobility and fragmentation significantly affect people and their interaction with their states, and thus the social contract. Mobility is crucial in allowing people to explore opportunities beyond state borders and thus essential for their self-determination and well-being, but also is a source of unease *vis-à-vis* equal concern and respect, including distributive justice. Fragmentation, in turn, offers *à la carte* menus that enable some taxpayers to tailor public goods and services more specifically to their preferences. Though possibly helping to maximise satisfaction of their preferences and promoting their self-determination, fragmentation often exacerbates the potential harms of mobility. If taken to its extreme, fragmentation challenges the system’s very structure by endorsing a new type of affiliation—a non-subscription option to interact with various states while being a member of none. The availability of this non-subscription option, far more than providing taxpayers with more (and more nuanced) choices, threatens the traditional system of sovereign states formed around membership in a political community.

Together, mobility and fragmentation not only limit the ability of states to collect the taxes needed to provide necessary public goods and secure justice, but could also undermine the social contract by imparting an itemised, marketised and short-term *quid pro quo* stance on membership and on the provision and consumption of public goods and services, an approach that corrodes the co-authored rationale of the contract.

Despite its many discontents, however, globalisation is not the enemy here. Economic globalisation, which has generated unprecedented growth and prosperity for capital owners, has also proved to be a reasonable deal for many poor workers in many countries. Even more significantly, globalisation’s expansion of opportunities, together with its checks on state power, is crucial for people’s liberty, allowing them to satisfy their preferences and pursue their goals.

And yet, the challenges that mobility and fragmentation pose to the social contract and thus to international taxation are fundamental and require us to rethink a social contract appropriate to an era of globalisation. Beyond the technical problem of designing taxing and spending systems for a global setting, we need to re-establish the social contract as a sustainable foundation underwriting the relationships between states and their constituents. The challenge, in other words, is to revitalise the very basis of the social contract: the ability of members in a political community to co-author their collective will. The new social contract should seek to preserve states’ just institutions and their ability to support their constituents’ collective self-determination without rolling back the greater opportunities people enjoy under globalisation. My goal in this article is to refine the considerable challenge entailed by this task by unpacking the issues at stake and presenting the essential dilemmas they involve.

This article proceeds as follows. Section 2 sets the stage by explaining the key features of the social contract in the domestic setting of a closed economy: the public provision of goods and services, the duties of equal concern and respect it entails, including distributive justice, and the nature of membership it demands in the political community of the state. Sections 3 and 4 discuss the predicament of social contracts under globalisation. Section 3 examines how exposure to the global market affects the social contract by studying the two factors of globalisation: mobility and fragmentation. Section 4 explains how these factors reshape the interaction between states and their constituents. After exploring the implications of these processes for the features of the social contract, it concludes that, if left unattended, mobility and fragmentation threaten to unravel the social contract as we know it. Section 5 concludes with some preliminary reflections on the way forward.

## 2. Tax and Citizenship: A Social Contract

The starting point for this discussion is the domestic arena: the interaction between the state and its members and taxation’s crucial role in this interaction. Taxes finance the state, allowing it to pay for the people’s joint projects and enabling them to pursue their collective self-determination through state institutions. Notably, taxes also offer a way (some argue the best way^2^) to promote distributive justice, thus ensuring the legitimacy of the state’s coercive system.

One way to explain this interaction is the social contract metaphor. The social contract is a loaded concept in political philosophy.^3^ Here, I use the concept as a metaphor describing a pact between a group of people seeking to co-author the instrument of collective self-determination created by the people and for the people to promote their collective will in a way that renders it both stable and beneficial over time.^4^ In this thought experiment, the state is entrusted with the authority to coerce the members of its political community so as to afford them goods and services of a greater scale and scope, as long as it treats all of them justly.

Under a well-functioning social contract, state institutions deliver greater stability and security. They facilitate people’s ability to make plans for the future with relative assurance that these institutions will comply with their obligations and enforce the duties of others. Ideally, state institutions allow members of this self-determined political community to live more fulfilling and less strenuous lives.

Taxation has a major role in upholding the financial aspects of this project. Through the fiscal system, we entrust the state with the monopolistic power to coerce us to pay for publicly provided goods and services and to decide which goods and services will be provided. In the ‘publicly provided goods and services’ category, I include not only pure public goods and services which the market could not adequately provide on its own due to collective action, externalities or high transaction costs (such as law and order), but also merit goods that the state provides in fulfilling its mission to enable and facilitate people’s well-being and self-determination (such as public healthcare), as well as goods and services that would not carry the same meaning if not pursued jointly (such as public education, art museums and public broadcasting).^5^

This state’s power to coerce people to pay taxes demands justification, implying that the state’s authority depends not only on its service to our collective goals, but also on its compliance with the demands of justice. Followers of traditional tax theory may find echoes of these tenets of state authority in the requirement that taxes must not only be efficient (to maximise welfare), but also equitable.^6^ For some, it is the coercive power of the state that demands justice.^7^ For others, rather than the coercion, it is the sheer involuntariness of the state’s rules that imposes a duty of justice. As Andrea Sangiovanni notes, the state

must give each of us a special reason to accept its laws strong enough to rebut any objection we might have to them. The justification, in turn, must show that the law could reasonably be seen as acceptable from within each person’s individual point of view, although no one consents to it.^8^

Acceptance requires the law to show equal respect and concern for all its constituents.^9^ As Ronald Dworkin put it:

A political community that exercises dominion over its own citizens, and demands from them allegiance and obedience to its laws, must take up an impartial, objective attitude toward them all … Equal concern … is the special and indispensable virtue of sovereigns.^10^

This duty of *justice* has two dimensions. One is *horizontal*: since taxation speaks on our behalf as co-authors of this social contract, it must treat all of us with *equal concern and respect*, which I claim is what tax jargon’s ‘horizontal equity’ truly means. The taxing state is obligated to treat us, rather than as abstract beings, in our more robust sense of personhood—to see us as we are.^11^ The tax system must heed, for example, that a person’s gender may imply greater responsibilities of care or that a person’s disability may imply extraordinary costs in generating income. Likewise, taxpayers who are less mobile globally should not be disadvantaged compared to those who are more mobile.^12^

The second dimension entailed by equal respect and concern is *vertical*. It requires the state to ensure the fair distribution of the social welfare pie.^13^ What exactly would be considered fair distribution for the social contract to be acceptable is, of course, debatable, but what is clear is that *distributive justice considerations* must be part of any tax debate in the domestic setting. Moreover, except for subscribers to strict libertarianism, most would agree that some level of redistribution is necessary. (Rawlsians would argue that it is hard to claim that stakeholders would not have agreed to it behind the veil of ignorance.)

Tax and justice are thus inherently entangled in the economic project of the state. The coercive power to tax enables the state to operate and allows for the public provision of goods. At the same time, the coercive power of the state demands justice. Justice—equal respect and concern, including at least some threshold of distributive justice—provides the coercive power of the state with its necessary legitimacy. Thus, taxes need not only support our collective welfare by financing public goods and services, but must also be just: they must treat each of us with equal concern and respect, including being distributively just.

The third key feature of the social contract justification of the state is the concept of *political membership*. The social contract metaphor is designed to explain that ‘members of some society have reason to endorse and comply with the fundamental social rules, laws, institutions … of that society’.^14^ Like the founding members of a club, a community or a corporation who declare themselves (as well as potential future participants) members and thus claim entitlement to certain rights and benefits and assume certain duties, the social contract metaphor envisions a *defined* group of members in the state’s political community who, by dint of their membership, subject themselves to the duties it imposes and claim the rights it confers.^15^

Political membership is obviously more than a consumers’ club. Member-constituents are not merely users of the goods and services the state provides, nor are they merely subjects of its coercive regime. Rather, as parties to the social contract, they are co-authors of the state’s regime. Their affiliation with the state is thus an expression of their *civic membership* in a political community.^16^ In Nagel’s terminology, they are part of a unique ‘coercive co-authorship’, or, as Joshua Cohen and Charles Sable explain, ‘individuals are both subjects in law’s empire and citizens in law’s republic’.^17^ The state’s political mechanism facilitates members’ co-authorship in constructing the bundles of goods and services it provides and determining the corresponding taxes. Thus, as the famous ‘no taxation without representation’ saying goes, tax is linked at its core to the idea of co-authorship in a political community. Members of this political community take up unique commitments to obey its rules and support its just institutions.^18^ They are thus entitled to the goods and services the state provides and owe their peers a duty of justice. These abstract maxims translate in the context of taxation into a duty to comply with tax rules that pursue just goals of horizontal and vertical equity.

In short, the social contract underpinning a political community’s tax system: (i) facilitates the *public provision of goods and services*; (ii) demands the tax system’s compliance with the requirements of *horizontal equity*; (iii) pursues *distributive justice*; and (iv) gives meaning to *civic membership in a political community*.

This analysis, seemingly straightforward in a domestic setting, becomes knotty in a globalised one, giving rise to the essential problem of international taxation: how to settle the tension between a state-based regime and a competitive global reality. I now turn to the potential effects of globalisation (specifically to two of its key aspects—mobility and fragmentation) on the features of the social contract in the context of taxation.

## 3. Two Features of Globalisation

### A. Mobility

Under globalisation, people increasingly relocate in search of places they view as serving their goals and interests. People’s mobility drives (and is driven by) competition between states seeking to attract talented, productive, young and wealthy individuals able to contribute to their economy, their culture or even their social security systems.^19^ In competing for in-demand residents, states offer them attractive packages of public goods and services catering to their needs and preferences.^20^ Taxation could be part of the appeal, attaching an enticing ‘price tag’ to the sought-after bundles of public goods and services. When relocating, people exchange one bundle for another, but the concept of membership in a given political community remains a key feature of the system. Indeed, much of the international tax system is premised on this concept of membership, where states base a large part of their tax regime on people’s personal nexus to their state.^21^

Competition subjects states to market supply and demand, potentially affecting the ‘price’ of the goods and services they provide as well as the mix, quality and quantity of the goods and services they offer. States are more likely to have an incentive to offer goods and services that mobile individuals find attractive and to reduce their tax burden in order to make their ‘prices’ competitive. These competitive forces may thus end up limiting the level of taxes collected by states. As competition intensifies, it may also affect the kinds and levels of public goods and services states provide (in line with the preferences of mobile individuals). Intense competition may even affect the size and constituency of the group subject to tax as a function of the attraction that certain bundles of public goods and services hold for various groups of taxpayers.^22^

The *temporal dimension* of people’s mobility merits particular attention here. Often, there is a time lag between people’s consumption of publicly provided goods and their tax payments. If people’s residency changes between these two events, what the state was expecting to collect in tax revenues may not match what it does collect. The mismatch is manifested in (at least) two ways.

First, it is not uncommon for people to retain options to go back and use the public goods and services offered by their original states (eg their citizenship, their right to work or their eligibility for public health or old-age benefits) and choose to exercise their right to use them only if and when they need to (eg when they get older or when they seek vaccinations or public funds made available during the pandemic). In these circumstances, people benefit from the state’s past investments in support of health and education, which are designed to create positive externalities, or from its risky investments in public goods, such as supporting basic science or countering a pandemic.

Second, people who benefited from public goods in the past may later opt to reside, incorporate or generate their income elsewhere by choosing to work overseas.^23^ The situation becomes particularly acute when emigration and immigration levels are asymmetrical, as is often the case in less developed countries, which tend to lose members of their affluent and educated elites who are in high demand elsewhere without the redress of immigration.

The mismatch between taxing people’s life-long ability and their consumption of publicly provided goods and services interferes with the ability of states to internalise the benefits of long-term investments in public goods or projects that the state and its citizens may find important. Thus, for example, if a state supports higher education on the assumption that educated individuals will earn more and thus pay more taxes, but these individuals end up leaving the country to pay taxes elsewhere, their home state stands to lose.

### B. Fragmentation

Although fragmentation is a concept less familiar than mobility in the literature on international taxation, it is a key feature of the system. Fragmentation markedly changes the analysis by defying the comprehensive conception of membership in a political community. It unties the bond between membership and entitlement to state-provided goods and services on the one hand and taxation on the other. Under fragmentation, (some) taxpayers can unbundle the packages of goods and services that states offer and consume them *à la carte*.^24^ Rather than commit, for a certain tax price, to the shrink-wrapped package offered by a single jurisdiction, that of their membership, sophisticated taxpayers can pick and choose from goods and services offered by different jurisdictions for a relatively modest tax ‘price’. In the extreme manifestation of this phenomenon, they can even opt out of membership itself.

#### (i) Public goods à la carte

While the competition for members generated by mobility requires individuals to make a binary choice—either staying and subscribing to the domestic bundle or leaving and subscribing to another—unbundling opens up a host of attractive alternatives. Fragmentation thus allows some people to *diversify* their interactions with states, that is, to simultaneously consume goods and services offered by multiple jurisdictions (often paying for each of them separately), including the use of selected regulatory regimes. Thus, for example, they can subscribe to the contract laws of New York and use the UK court system by the terms of their own contracts. They can use the corporate governance regime of Delaware simply by incorporating there.^25^ They can enjoy the innovative regulatory environment of Singapore^26^ or use the securities regulations of the United States by trading on the US exchange.^27^ They can even adopt a child or hire a surrogate mother in a foreign jurisdiction when their home country does not allow that.^28^ They can study in publicly supported institutions overseas, invest in a thriving high-tech industry cultivated by public investment or work in a prosperous labour market in a foreign jurisdiction where their talent or creativity is in high demand.

As mentioned earlier, this diversification is facilitated by states offering some of their goods and services *à la carte* to non-members for a fee (as in the case of incorporation fees^29^) or for resources they seek such as FDI,^30^ the human capital of top athletes or scientists^31^ or R&D.^32^

Membership, on the one hand, and day passes (or permits to use facilities), on the other, represent the two poles on a spectrum of possible modes of interaction with the state. Membership represents a full subscription, entitling its holder to the full range of rights a state provides and imposing all the duties it requires. Day passes, by contrast, grant only a limited right to use a given set of goods and/or services provided by the state. Presumably, users of such goods and services *à la carte* will only purchase them if the price is right. States are expected to offer these goods and services to occasional users (only) when the marginal cost of offering them is lower than their benefits.

The use of publicly provided goods and services *à la carte* is largely facilitated by *choice of law* rules, which allow people some leeway in selecting the rules that will apply to them, irrespective of their other co-ordinates.^33^*Technological capacities* also allow taxpayers to enjoy goods and services publicly provided by various regimes. People can benefit from publicly supported educational institutions overseas while studying online, they can work remotely and enjoy the labour market standards (and sometimes the legal protection of) other jurisdictions and they can use the (foreign-regulated) banking system, financial or insurance industries in other jurisdictions by engaging with them remotely. While some jurisdictions try to limit the application of their legal regimes on a territorial basis, others do not insist on such restrictions.^34^

Choice of law mechanisms, technological developments and the temporal mismatch discussed above untie the links between people’s membership in a state (and the membership fees it imposes) and their consumption of the goods and services it provides. States providing these goods and services do not impose taxes based on people’s ability to pay, and certainly not on their life-long ability to pay. Where non-residents are concerned, states can, at best, collect taxes or fees that reflect the benefits to non-resident users or profit from the positive spillovers of their activities. Competition may lead states to settle for a reduced *quid pro quo* when the marginal costs of providing goods and services are lower than the benefits. Ultimately, people who can consume publicly provided benefits overseas will often be able to purchase superior services at lower costs.

#### (ii) Non-subscription options

A growing number of jurisdictions offer a complimentary feature beyond *à la carte* menus, which allows taxpayers not only to choose the goods and services they wish to consume, but also to avoid paying for the ones they do not. These jurisdictions offer a limited tax base for a special category of residents—what I call a ‘non-subscription’ option. Non-subscription options allow taxpayers to reside in jurisdictions without paying membership fees. Beyond their recourse to many of the public goods and services offered by their newly adopted hosts and their *à la carte* purchases of such goods and services in other jurisdictions, non-subscription offers these individuals a qualitatively different alternative as well: absent non-subscription, purchasing goods and services *à la carte* still requires purchasers to pay for similar goods and services they do not consume in their original jurisdiction of residence (assuming it does provide such services). By contrast, individuals who reside in non-subscription regimes and supplement them with goods and services purchased in other jurisdictions can pay solely for goods and services they wish to consume without paying subscription fees to any jurisdiction.

A famous example is the UK non-domiciliary or ‘non-dom’ regime, which allows eligible people to reside in the UK without paying the membership fees it usually charges, that is, without paying worldwide taxes based on their ability to pay.^35^ Residents eligible for this non-dom status can effectively shield their foreign-source income from UK taxes and instead pay a ‘fixed charge’ of £30,000–60,000.^36^

More specifically, the UK non-dom regime prescribes that the foreign income and capital gains of individuals resident but not domiciled in the UK are only taxed if remitted (brought back) to the UK. To be eligible, taxpayers must be domiciled overseas and have been living in the UK for either 7 of the last 9 years or 12 of the last 14 years.^37^ Eligible individuals can opt for the ‘remittance basis’, under which their foreign income and capital gains are subject to UK tax only if remitted to the UK. Since individuals can independently decide whether to bring their income and gains back to the UK or not, the rules allow them to effectively exempt their overseas income and gains from UK taxation, and pay instead an annual remittance-basis charge.^38^ Taxpayers who satisfy the 7-year test pay £30,000; those who satisfy the 12-year test pay a higher remittance basis charge of £60,000.^39^ Either way, this regime is beneficial only to people who have substantial wealth abroad.

In what seems like a strategic move designed to attract high-net-worth individuals, other countries, such as Italy,^40^ Malta,^41^ Ireland (another domicile-based remittance scheme),^42^ Israel (a new residents exemption scheme)^43^ and Portugal (a preferential regime for ‘non-habitual’ residents),^44^ offer similar tax regimes. The relatively long-term residency requirements of these non-subscription schemes allow people to disconnect from their previous residency status. Thus, people can opt out of the social contracts system altogether and relate to *all* states as mere providers of goods and services *à la carte*.^45^

## *

In sum, in the wake of mobility and fragmentation, states can no longer be conceptualised as completely separate political communities, each collectively providing bundles of goods and services as part of their distinct co-authored projects. As the borders of these communities become porous and flexible, people can and do cross them physically and virtually along many dimensions, and states may find themselves competing along multiple margins, vying for residents and their activities in terms of time and scope for physical as well as virtual participation. This hyper-competitive reality is very different from the vision of the state in the traditional story of the social contract we started from.

## 4. Can Social Contracts Survive Globalisation

How do mobility and fragmentation affect the ability of states to publicly provide goods and services? Specifically, how do mobility and fragmentation affect the social contract’s normative underpinnings? To examine this question, we need to evaluate the implications of mobility and fragmentation for efficiency, distributive justice, horizontal equity and civic membership, but first for people’s liberty and self-determination.

### A. Liberty and Self-determination

People’s liberty and self-determination are at the very core of this discussion, both constituting the crux of the social contract’s rationale and supporting people’s mobility and freedom to choose where and how to operate. By sustaining state institutions, the social contract enables people to activate their self-determination in ways they could not otherwise pursue. While mobility allows them to choose their affiliations, fragmentation gives them a chance to seek opportunities that may be lacking where they reside without forcing them to leave and provides them with a plurality of options beyond what a single jurisdiction can reasonably be expected to offer. My starting point, then, is that mobility and fragmentation are essential for people’s liberty.

Mobility entails a power of exit that further protects individual liberty against the government’s power to treat minority stakeholders unfairly.^46^ People free to select their affiliations can better fulfil their own autonomous goals,^47^ helping to create communities they have actively chosen to be part of. Fragmentation further expands people’s options beyond national borders. Diversifying their consumption of publicly provided goods and services gives them a chance to seek opportunities that may be lacking where they reside without forcing them to leave (eg superior higher education or adoption opportunities less accessible in their country of origin). Together, mobility and fragmentation offer people alternatives to their ways of life, promoting their liberty and self-determination.

But these happy consequences are not available to everyone. Moreover, the exit options of mobility and non-subscription open to some may undermine the self-determination of those left behind. First, to be viable, some public goods must be provided on a large scale. If demand for certain goods and services declines due to the exit of some constituents, states might be unable to offer certain public goods or be forced to lower their quality. Second, long-term planning, a key element of self-determination, frequently necessitates relying on the resources and support of fellow members. The fact that some members of the community might opportunistically leave may pull the rug from under the feet of those left behind. Finally, anticipating the departure of mobile individuals, states may be less inclined to invest in long-term goals and thus be less able to support the long-term plans of their constituents. Thus, supporting the unrestricted liberty of mobile individuals could undermine the ability of other community members to flourish.

The fact that some people may be left behind with no option of relying on their peers for their long-term plans need not require the imposition of limits on people’s mobility or their right to seek opportunities elsewhere. Presumably, the solution should be to broaden the range of opportunities available to those lacking them rather than limit the liberty of others in their community. And yet, even if similar liberties were made available to everyone, some people will not be able to benefit from them. For example, vulnerable individuals may be unable to exploit better opportunities elsewhere, and the option of relocation or consuming services overseas is thus of little or no real benefit to them.

### B. Efficient Public Provision of Goods and Services

The social contract, as noted, is designed to allow governments to provide public goods and services that the market alone could not adequately provide, including pure public goods, merit goods, and goods and services that, unless pursued jointly, do not carry the same meaning. In closed economies, the state’s coercive power enables it to provide such goods and services even when the market alone cannot.

Mobility and fragmentation can have positive effects on the efficiency of states’ public provision of goods and services. Consider mobility first. As the Tiebout model explains, a situation of people ‘voting with their feet’ reveals information that helps to bridge the gap between taxpayers’ preferences and the kinds and levels of public goods supplied by governments.^48^ According to the model, when perfect mobility exists, citizens can select among the bundles of public goods and services offered by various jurisdictions with tax ‘price tags’ attached. Though not perfect, this selection process increases the taxpayers’ choice-making capacity and, thus, their preference satisfaction. The pressure of competing for residents also helps improve efficiency in providing public goods and services.

Fragmentation pushes Tiebout’s consumerist logic to its extreme. The ability to pick and choose from the unbundled goods and services provided by various jurisdictions offers more nuanced choices and presumably increases preference satisfaction, where taxpayers can tailor the goods and services they consume to fit their preferences. Governments can thereby also acquire more refined information about the demand for specific goods and services, allowing them to specialise in high-demand areas where they hold a comparative advantage. States offering *à la carte* services or non-subscription regimes can presumably gain from the expanded clientele, as well as from attracting mobile taxpayers bringing relevant skills and resources insofar as the benefits they offer the state, including whatever they pay, are greater than the marginal costs of the services they consume.

That said, globalisation may entail considerable downsides potentially detrimental to efficiency. First are the usual critiques against tax competition. Strategic competition could drive tax rates down to a suboptimal level, forcing states to under-provide public goods.^49^ Externalities, free-riding and tax avoidance undercut even further the ability of states to optimise the provision of public goods and services.

Second, even absent these market failures, efficient provision of some publicly provided goods requires some scale, and if too many people opt for a non-subscription option, some states may be unable to engage in large-scale projects.

Finally, the temporal dimension raises particularly upsetting concerns for a special subset of publicly provided goods and services—those designed for the long term. When the time and place at which income is taxed and benefits are consumed are mismatched, the social contract is destabilised. Unable to internalise the (tax and other) benefits from their long-term investments, states lack incentives for supporting activities that might yield benefits in the future.

In sum, the efficiency effects of mobility and fragmentation are inconclusive. On the one hand, they may improve preference satisfaction and the states’ efficient provision of goods and services. On the other hand, market imperfections and a bias against large-scale projects and long-term commitments undermine efficiency in publicly providing goods and services.

### C. Distributive Justice

Both sides of the social contract—the goods and services provided by the state and the taxes paid—implicate distributive justice: to redistribute wealth, the government can either collect more from the rich or provide superior benefits to the poor (or both). Mobility and fragmentation thus affect both sides of this equation.

Consider mobility first. At the most basic level, mobility pressures states to compete, leading them to lower their taxes, thus reducing the resources needed to support their welfare systems. The lower the taxes a state collects, the lower the level of goods and services it can afford to publicly provide.

Moreover, the pressure to reduce taxes may affect taxpayers differently, potentially undermining the system’s progressivity. Some people are more mobile than others. Talented, wealthy and young individuals presumably enjoy greater flexibility in their choices of residency, not only because they have better skills and more resources, but also because, as mentioned above, they are often in high demand in countries that tend to offer their like easy access (while blocking entry to others). Thus, some people’s decision to relocate is presumably more sensitive to their tax burden. The more elastic their decision and the greater their tax burden, the greater the chance they will decide to relocate.^50^

Given these individual differences, states have an incentive to ‘price discriminate’ among taxpayers, taxing mobile and desirable taxpayers more leniently than immobile and less ‘attractive’ ones. Similarly, the goods and services states offer may be tilted in favour of the more appealing taxpayers in an effort to retain them or attract new ones: the quality and quantity of goods and services that they are less interested in may decline and, in turn, those that disproportionally interest them may increase, resulting in a bundle of publicly provided goods that caters to the preferences of mobile taxpayers.

On the assumption that the rich are more mobile than others, the need to increase their taxes in order to reduce inequality is in tension with the pressure to lower their taxes to retain or attract them. Hence, not only do the available funds shrink due to the tax competition between states, but the allocation of the tax burden as well as the selection of goods and services might change in favour of in-demand taxpayers, who are precisely those that states need to tax more heavily in order to redistribute.

*À la carte* consumption, and non-subscription regimes in particular, hinder even further the attempts of states to use the tax system to reduce inequality. When individuals are tempted to relocate to non-dom regimes, they can pay no membership fees, and instead consume only the kind and level of goods and services they need or want, and get them at a low cost in foreign jurisdictions. That is, by moving into a non-subscription jurisdiction, such individuals do not have to give up all the upper-scale goods and services provided by some states. Instead, they can choose to reside in a low tax–low service jurisdiction and consume superior goods and services *à la carte* elsewhere. Moreover, because they do not pay membership fees anywhere, the services they do not wish to consume might lack funding. This further limits what the state has to offer to those left behind. Thus, less-mobile individuals might have to put up with an impoverished version (if any) of the goods and services their government provides.

Jurisdictions offering non-subscription options, therefore, degrade the social contract in *other* jurisdictions. If these arrangements become available in a critical mass of countries, *all* states may find it unfeasible to promote distributive justice through their tax systems. Moreover, if such arrangements become prevalent, it might further broaden the gap between the ones capable of leveraging on competition and those who are left behind. The non-subscription option allowing wealthy taxpayers to reside in non-dom jurisdictions and shielding their personal tax base from the jurisdiction of any state thus denigrates the social contract.

### D. Horizontal Equity

Recall that the requirement to justify the social contract has two dimensions. Alongside the vertical dimension of distributive justice, justice demands some form of horizontal equity among the members of the pertinent political community, requiring the state to see us as we are rather than as abstract beings. The global competitive scene, however, may alter states’ perspectives, highlighting people’s use value and thus undermining their value as equals. State power may increasingly shift from a coercive authority controlled by people’s political will to a give-and-take interaction ruled by market forces, where supply and demand reign and exit prevails over voice. A reality of competition urges policy makers to ‘think like firms’^51^—to consider factors that can maximise the domestic advantages accruing from current and potential taxpayers while minimising costs. Mobility may push them to pursue ‘valuable’ taxpayers, especially the more elastic ones, meaning those more inclined to relocate to obtain superior ‘deals’ of public goods and services for lower taxes. Unfortunately, this marketised perspective may incentivise states to invest less effort in retaining members who lack any realistic option to move since other countries find them less attractive.

Mobility’s uneven distribution affects the notion of equal respect and concern for all. By focusing on people’s attractiveness as members and on the elasticity of their relocation decisions while offering desirable bundles of public goods and services for a tempting price, mobility intensifies their use value rather than their status as equal members in a political community entitled to equal concern. This is true for mobile individuals who are valued as a function of their exit power and even more true for those left behind, who, rather than being viewed as equal members, are reduced to their (lack of) mobility value. Treating some individuals differently simply because they—unlike others—have lesser options to leave does not comply with equal respect and concern. Such individuals face a potentially worse situation if they also belong to a group otherwise disadvantaged, which is the case with many vulnerable groups. And the picture becomes more complex when the *mobile* are members of vulnerable minority groups treated unequally by the state. When these groups’ political voice is not heard, their exit power could serve to amplify it, as Albert Hirschman has explained,^52^ and may even lead to their equal treatment.

Unlike mobility, allowing constituents to consume additional public goods and services provided *à la carte* by overseas jurisdictions does not seem to undermine horizontal equity.^53^ As long as they pay their taxes at home, not only can their government still finance the bundles of public goods and services that supply the needs of others in the community, but their recourse to multiple jurisdictions may even help to cater to some specific needs and alleviate inequalities that their home jurisdiction fails to accommodate. Thus, for example, where domestic institutions lack resources to make higher education accessible to people with disabilities, foreign institutions may be a viable alternative. This arrangement, however, does not solve the problem of their own states ignoring their unique traits or needs.

The ability to pick and choose the providers of public goods and services, however, is often contingent on economic and social resources that are not allocated equally. Hence, having states rely on other jurisdictions to provide services essential for people’s self-determination could be detrimental to the equal treatment of those lacking access to foreign sources.

### E. Civic Membership in a Political Community

A political community of equals requires, as noted, a social contract where all members can be taken to be co-authors. Rather than merely an aggregation of individual members’ preferences, the social contract entails a process aimed at formulating a common set of often incommensurable normative goals that citizens’ long-term commitments converge around, prioritise and translate into a set of publicly provided goods and services that promises to outlive its members. Mobility and fragmentation have a mixed effect on civic membership. Three points need to be addressed.

First, as noted, the exit options provided by mobility and fragmentation keep a healthy check on the power of the majority. The availability of other communities and other options for consuming public goods and services elsewhere pushes governments to be more responsive to the people’s voice.^54^ Voice often has little effect in the absence of a credible exit threat; thus, exit supports civic membership.

But people do not all enjoy equal exit options, and those with better ones exert greater influence over the political process. Putting it bluntly, when people have less exit power, their voice may count less. Exit, then, largely prevails over voice, limiting the community’s ability to pursue its collective goals. Again, however, the problem focuses on the distribution rather than the existence of exit options. Were the choice to leave and the choice to consume public goods elsewhere allocated equally among all members of society, mobility and fragmentation as such would not pose such a serious problem for civic membership.

Second, as often happens when market norms infiltrate other spheres, consumerist self-interest considerations may crowd out other commitments. Members may view public goods and services as mere commodities, satisfying their preferences irrespective of who fulfils them or who else is entitled to them. The significance of collective decision making is downplayed when fragmentation helps these individuals attend to their needs separately from their co-constituents, and mobility guarantees that, if necessary, they will be able to join another political community.

Third and last, providing people with a range of options to select from, mobility and fragmentation may have complex effects on their loyalty, namely their commitment to their community. Because people can opt out, their commitment to their community becomes somewhat tentative. Choice is crucial for the liberty of those who can exert it and, in itself, is not necessarily a problem for civic membership. Indeed, staying could become more valuable when a viable option to leave is available. Their decision to stay, then, rather than a default option, is essentially a choice to be a part of their community, strengthening their civic commitment. The very costs imposed on people who choose to give up better opportunities elsewhere add to their credibility and to that of the political discourse.

At the same time, mobility opens up possibilities for *opportunistic* exits that may prove harmful to civic membership. Opportunistic behaviour undercuts members’ long-term expectations and frustrates their ability to rely on their political communities to support their long-term plans. Constant concern about opportunistic moves by others undermines collective self-determination and potentially downgrades civic membership.^55^

In sum, we tread a fine line between the costs and benefits of mobility and fragmentation on the one hand and civic membership on the other. Free movement and availability of options to choose from are key features of a thriving political community. The risk lies where a certain threshold is crossed, so that exit completely crowds out voice, and a completely marketised and fragmented competition emasculates the very existence of political communities and thus of civic membership.

## 5. The Way Forward

I will conclude with a reflection on the way forward: refining the mission at hand, explicating the policy options available to states for approaching this mission unilaterally and envisaging a possibly utopian global co-operative accord that reinstates social contracts for all.

In section 4 above, I noted that there is a fine line to tread between the advantages and disadvantages of mobility and fragmentation for liberty, efficiency, distributive justice, horizontal equity and civic membership. This may seem too multifaceted and complex in order to yield concrete practical takeaways. But in fact, the discussion boils down to two recurring challenges for the social contract under globalisation. Designing a social contract in an era of globalisation demands that we: (i) settle the asymmetry between those who benefit from mobility and fragmentation and those who do not; and (ii) ensure it meets this task without undermining people’s liberty and opportunities to pursue their goals.

On its face, meeting both requirements seems unfeasible. Individuals and political communities confront a seemingly irreconcilable clash between two deep normative commitments: to prioritise individual liberty, highlighting personal choices and preference maximisation, or to prioritise commitment to others, restraining the choices available to individuals in order to support their communities’ collective will.

Although this tension cannot be entirely defused, tax law can ameliorate it. Taxation is the mechanism that can help to care for those who cannot make use of greater liberties without barring people from doing what they wish to do. This, in fact, is the financial essence of the social contract: to collect taxes from those with greater means in order to publicly provide goods and services to all. What makes tax unique is that it is the one legal instrument that allows us to collectively discharge our duties toward the have-nots without instructing people on what they should or should not do.

Mobility and fragmentation should thus be understood as introducing additional rifts demanding society’s attention. These rifts, like other categories of inequality relevant for tax purposes, present features that need to be addressed by tax law to resolve these rifts not only within states, but also *between* them.

Consider first the domestic level. Some states *can* address this task unilaterally. States that are attractive enough, with a constituency that is loyal enough and a membership regime that is ‘sticky’ enough, can probably enforce personal-based taxation (membership fees) since most of their constituents are unlikely to opt out for tax reasons. Thus, states in greater demand *can* presumably (unilaterally) stick to a relatively robust social contract by mandating full subscription taxes based on members’ ability to pay.^56^ One example of a jurisdiction that follows this path is citizenship-based taxation in the United States, imposed (even) on the worldwide income of its non-resident citizens.^57^

In sharp contrast, less attractive states with a constituency that is more elastic and a membership that is easier to shake off might be less resilient and feel continuously threatened with the loss of their strongest taxpayers.^58^ Especially if non-subscription options become prevalent, these states may be pushed to offer increasingly thinner levels of public goods and services, eventually turning the least attractive among them into no more than nominal clubs. These states might end up with no meaningful co-authored projects, with only a very thin layer of publicly offered goods and services, without any significant ability to address distributive injustices and thus also with an impoverished version of civic membership.

As for the inter-state level, resolving these asymmetries demands unprecedented co-operation and raises serious questions as to the scope of global justice. Based on previous co-operative efforts of international taxation, one could imagine possible co-operative tracks. States could, for example, co-operate in *targeting ‘harmful’ residency regimes* or *establishing a cartel of ‘minimal bundles’ or minimal tax rates*. Such regimes echo past attempts to control tax competition, such as the failed effort to fight ‘harmful tax competition’ and the—perhaps more successful, but still too new to evaluate—attempt to establish a minimum corporate tax.^59^

The latter is an admittedly wild idea, presumably designed to set a floor to competition—to allow all states to collect some taxes from the more elastic segments of their respective societies and to decide on some minimal packages of publicly provided goods and services required to preserve their social contracts. This is likely to be a heroic effort which demands separate analysis, carefully matching nuanced solutions to complicated problems. To name but a few: what might be an adequate minimum level of publicly provided goods and services and/or taxes? How can poor states be supported in offering adequate public goods and services? How can all this be achieved without overly infringing on states’ sovereignty? And are these achievements at all plausible given the foreseeable strategic behaviour and the expected pushback from numerous actors?

Be that as it may, the current discussion points to one specific pathology that this global effort should certainly address: non-subscription options. Were non-subscription options eliminated, an important negative component would be dismissed, and people would have to stay or become members of some political community. Although some states would still be able to offer only lesser services, they would be providing the same services to *all* their members and would no longer be in the position of selling the ring-fenced regime that, as shown, undermines the system. Achieving this happy goal, however, is far from easy. Conceptually, a classic line-drawing question is involved: determining what states cross the line between legitimately hosting foreigners for limited periods on the one hand and offering them non-subscription options on the other. But even if this bridge is crossed, a significant practical challenge remains: attaining such an agreement necessitates pressuring states to refrain from offering such regimes to newcomers, notwithstanding the benefits that this practice entails for them.

## *

To conclude, opening the door to mobility and fragmentation is, for better or for worse, changing the way we do (and should) think about the social contract by transforming our perception of the tax state and forcing us to rethink the nature of the interaction between states and their constituents. Given the indispensable role of the social contract for people’s well-being and self-determination, it is crucial to protect its suitability to support justice and ensure the public provision of goods and services necessary for people to thrive. Some states can do so on their own. But many—probably most—cannot. For those states to reinvigorate their social contracts, a global pact is vital. However, the conditions necessary not only for signing such a global pact but also for making it equitable make this attainment far from obvious. Only time will tell whether states can live up to this challenge.

## Footnotes

1 I deliberately use the term ‘metaphor’ in order to distinguish my conception of the social contract from thicker understandings of this concept. See the text accompanying n 3. For the purposes of this article, adjudicating between various such conceptions is unnecessary.

2 Louis Kaplow and Steven Shavell, ‘Why the Legal System Is Less Efficient than the Income Tax in Redistributing Income’ (1994) 23 JLS 667.

3 Under the imaginary social contract people are seen as exchanging some of their independence for membership in the political system. This description, of course, is an oversimplification of the concept of consent to the social contract; for a general survey of the debate, see eg Jonathan Wolff, *An Introduction to Political Philosophy* (3rd edn, OUP 2015) 42–6.

4 Fred D’Agostino, Gerald Gaus and John Thrasher, ‘Contemporary Approaches to the Social Contract’ in Edward Zalta (ed), *The Stanford Encyclopedia of Philosophy* (Winter edn 2021) ch 3.1 <https://plato.stanford.edu/archives/win2021/entries/contractarianism-contemporary/#Consent> last accessed 7 September 2023.

5 See respectively John Black and others, *A Dictionary of Economics* (OUP 2009) 120; Richard Musgrave, *The Theory of Public Finance: A Study in Public Economy* (McGraw-Hill 1959) 13–15; Elizabeth Anderson, *Value in Ethics and Economics* (Harvard UP 1993) 158–63.

6 See eg Adam Smith, *An Inquiry into the Nature and Causes of the Wealth of Nations* (OUP 1976) Book 5, ch 2, 825–8; Liam Murphy and Thomas Nagel, *The Myth of Ownership* (OUP 2002) 12; Michael Graetz and Deborah Schenk, *Federal Income Taxation: Principles and Policies* (4th edn, Foundation Press 2002) 25–7; Reuven Avi-Yonah, ‘The Three Goals of Taxation’ (2006–07) 60 Tax L Rev 1, 3.

7 Thomas Nagel, for example, often refers to coercion as both ensuring co-operation ‘because it doesn’t take many defectors to make such a system unravel’ and, at the same time, requiring legitimation. Nagel contends that ‘The state makes unique demands on the will of its members … and those exceptional demands bring with them exceptional obligations, the positive obligations of justice’; see Thomas Nagel, ‘The Problem of Global Justice’ (2005) 33 Philosophy & Public Affairs 113, 116.

8 Andrea Sangiovanni, ‘Global Justice, Reciprocity, and the State’ (2007) 35(1) Philosophy & Public Affairs 3, 15; see also Rainer Frost, *The Right to Justification: Elements of a Constructivist Theory of Justice* (Columbia UP 2012).

9 As Nagel maintains, ‘states not only foster co-operation by coercively enforcing rules but implicate the will of those subject to their coercive authority by making, in the name of all, regulations that apply to them all’. Joshua Cohen and Charles Sabel further explain that such will-implication is significant since ‘it is impermissible to speak in someone’s name … unless that person … is … given equal consideration in making the regulations’. Rules imposed by the state must therefore be justified to their co-authors. ‘And not just any justification will do … the justification must treat each person … in whose name the coercion is exercised—as an equal.’ Joshua Cohen and Charles Sabel, ‘Extra Rempublicam Nulla Justitia?’ (2006) 34(2) Philosophy & Public Affairs 147, 160.

10 Ronald Dworkin, *Sovereign Virtue: The Theory and Practice of Equality* (Harvard UP 2000) 6.

11 There is some confusion, I claim, as to the term of horizontal equity when some scholars examine it strictly based on means; see eg Louis Kaplow, ‘Horizontal Equity: Measures in Search of a Principle’ (1989) 42 National Tax Journal 139; Louis Kaplow, ‘A Note on Horizontal Equity’ (1992) 1(3) Florida Tax Review 191 (conceptualising horizontal equity as simply one point on the spectrum of vertical equity). I argue, instead, that in order to treat each of us with equal concern and respect, horizontal equity must reflect many features or dimensions of people’s lives constitutive of their identities (eg gender, health, where they reside). For an analysis of these dimensions, see Tsilly Dagan, ‘The Currency of Taxation’ (2016) 84(6) Fordham L Rev 2537.

12 Dagan, ‘The Currency of Taxation’ (n 11).

13 Distributive justice has always been a key consideration in tax policy. See eg Henry Simons, *Personal Income Taxation* (University of Chicago Press 1938) 18–19; Murphy and Nagel (n 6) 12; Linda Sugin, ‘Theories of Distributive Justice and Limitations on Taxation: What Rawls Demands from Tax Systems’ (2004) 72 Fordham L Rev 1991.

14 D’Agostino, Gaus and Thrasher (n 4) ch 1.1.

15 Tsilly Dagan, ‘Klaus Vogel Lecture 2021: Unbundled Tax Sovereignty—Refining the Challenges’ (2022) 76(7) Bulletin for International Taxation 1.

16 Their membership in the political community allows them to create and be part of a larger social project. Membership represents a personal affiliation between the state and its stakeholders. People need political communities not only to attain various material or other ends, but also for identity and self-reference purposes. For members, the political community is a means of satisfying the human need for social stability and belonging. See Joseph Raz, *Ethics in the Public Domain: Essays in the Morality of Law and Politics* (OUP 1994) 74–155; Chaim Gans, ‘The Liberal Foundations of Cultural Nationalism’ (2000) 30(3) Canadian Journal of Philosophy 441.

17 Cohen and Sabel (n 9).

18 John Rawls, *A Theory of Justice* (Harvard UP 1971) 293–4.

19 There are, of course, other groups of people that are less fortunate: not only are they not sought after, but often they are actively rejected or oppressed by states. The discussion of their proper treatment, given the diverse reasons that motivate it—economic, social, political or humanitarian—implicate different sets of considerations and should thus wait for another day.

20 See eg Tsilly Dagan and Talia Fisher, ‘State Inc.’ (2018) 27(3) Cornell JL & Pub Pol’y 661 and references therein. See also Ayelet Shachar, ‘Citizenship for Sale?’ in Ayelet Shachar and others (eds), *The Oxford Handbook of Citizenship* (OUP 2017) 789, 790–1; Ayelet Shachar, ‘The Race for Talent: Highly Skilled Migrants and Competitive Immigration Regimes’ (2006) 81 NYU L Rev 148, 150. For some recent empirical work, see eg Eloi Flamant, Sarah Godar and Gaspard Richard, ‘New Forms of Tax Competition in the European Union: An Empirical Investigation’ (EU Tax Observatory, November 2021) Report No 3, 9–18 (noting that all schemes created to attract new taxpayers, with the sole exception of the UK’s remittance basis scheme, successfully attracted an increasing number of taxpayers); OECD, *Taxation and Employment* (OECD Tax Policy Studies 2011) 128–9.

21 There is strong academic consensus that a ‘sufficient nexus’ is required for a state to impose tax in a manner compliant with public international law. See Ajay Kumar and Asif Hasan Qureshi (eds), *The Public International Law of Taxation: Texts, Cases and Materials* (Kluwer Law International 1994) 44–65; Jacinto Martha, *The Jurisdiction to Tax in International Law: Theory and Practice of Legislative Fiscal Jurisdiction* (Kluwer 1989) 12–18.

22 Tsilly Dagan, *International Tax Policy: Between Competition and Cooperation* (CUP 2018) 43–71.

23 See Jagdish Bhagwati, ‘Taxing the Brain Drain’ (1976) 19(3) Challenge 34. Subsequent work has complicated the picture presented by Bhagwati, including through the suggestion that the return of highly skilled emigrants is sufficiently prevalent to have a net-positive effect on countries of origin: Oded Stark, Christian Helmenstein and Alexia Prskawetz, ‘A Brain Gain with a Brain Drain’ (1997) 55 Economic Letters 227. For a review of the relevant literature, see Jean-Christophe Dumont and Georges Lemaître, ‘Beyond the Headlines: New Evidence on the Brain Drain’ (2005) 56(6) Revue Économique 1275.

24 Dagan, *International Tax Policy* (n 22) 3.

25 The corporate governance rules of Delaware apply to corporations incorporated within the jurisdiction; see Eric Talley, ‘Corporate Inversions and the Unbundling of Regulatory Competition’ (2014) 101 Va L Rev 1649.

26 See Christopher Chen, ‘Regulatory Sandboxes in the UK and Singapore: A Preliminary Survey’ in Mark Fenwick, Steven van Uytsel and Bi Ying (eds), *Regulating FinTech in Asia: Global Context, Local Perspectives* (Springer 2020) 11.

27 US securities laws generally apply only to securities sold on a US exchange or to companies with a significant economic presence in the country; see Chris Brummer, ‘Post-American Securities Regulation’ (2010) 98(2) CLR 327, 336.

28 Both practices are increasingly restricted by unilateral regulation, but remain widespread: Elizabeth Bartholet, ‘International Adoption: The Human Rights Position’ (2010) 1 Global Policy 91; Katherine Voskoboynik, ‘Clipping the Stork’s Wings: Commercial Surrogacy Regulation and Its Impact on Fertility Tourism’ (2016) 26(2) Ind Int’l & Comp L Rev 336. For a recent review of different regulatory approaches to surrogacy with a particular focus on cross-border surrogacies, see Valeria Piersanti and others, ‘Surrogacy and “Procreative Tourism”. What Does the Future Hold from the Ethical and Legal Perspectives?’ (2021) 57(1) Medicina 47.

29 See eg Delaware Department of State, ‘Division of Corporations Fee Schedule’ (1 August 2023) <https://corp.delaware.gov/fee/> accessed 7 September 2023.

30 It is very common for countries to offer generous investment incentives: World Bank, *Fiscal Incentives for Investment and Innovation* (OUP 1995). See also Dagan and Fisher (n 20) 681–2. At the extreme end of the spectrum lies ‘investment citizenship’: see Lior Erez, ‘A Blocked Exchange? Investment Citizenship and the Limits of the Commodification Objection’ in Dimitry Kochenov and Kristin Surak (eds), *Citizenship and Residence Sales: Rethinking the Boundaries of Belonging* (CUP 2023) 335.

31 See Ayelet Schachar, ‘Picking Winners: Olympic Citizenship and the Global Race for Talent’ (2011) 120 Yale LJ 2088.

32 Many countries offer generous subsidies for R&D: see OECD, *OECD Science Technology and Industry Outlook 2023* (OECD 2023) esp chs 1 and 2.

33 Katharina Pistor, *The Code of Capital* (Princeton UP 2019) 68–9, 134–7. A straightforward example, and the primary choice-of-law rule in respect of contracts in the jurisdictions of the UK, is art 3(1) of the Rome I Regulation (Regulation 593/2008, now retained in EU law under SI 2019/834), which begins, ‘A contract shall be governed by the law chosen by the parties’. For a discussion of how private international law processes can feed into the fragmentation of tax sovereignty, see Tsilly Dagan, ‘The Global Market for Tax and Legal Rules’ (2017) 21 Florida Tax Review 148, esp 177–97.

34 Obviously technology does not operate in a regulatory vacuum, and thus these technological innovations are not devoid of law. But law often lags behind technology. See Gary Marchant, ‘The Growing Gap Between Emerging Technologies and the Law’ in Gary Marchant, Braden Allenby and Joseph Herkert (eds), *The Growing Gap Between Emerging Technologies and Legal–Ethical Oversight: The Pacing Problem* (Springer 2011) 19. These concerns have been dramatically exacerbated by recent developments to artificial intelligence: Lord Hodge, ‘Law and Technological Change’ (British Irish Commercial Bar Association, 4 April 2019) 12–14.

35 Income Tax Act (ITA) 2007, pt 14, ch A1 (ss 809A–809Z10).

36 ITA 2007, s 809C(4).

37 Even if the taxpayer never acquires a UK domicile of choice, the remittance basis is lost when the taxpayer acquires a deemed domicile on the beginning of their 15th year of UK residence in the last 20 years (ITA 2007, s 835BA(4); Inheritance Tax Act 1984, s 267(1)). It must be remembered, however, that the protection of trusts settled before the acquisition of a deemed UK domicile enables the wealthiest taxpayers to keep much of their estate permanently outside of the UK tax net.

38 ITA 2007, ss 809H and 809C(1A). Another consequence of claiming non-domiciled status is that the taxpayer will lose entitlement to any tax-free allowances for income tax (ITA 2007, s 809G(2)) and capital gains tax purposes (Taxation of Chargeable Gains Act 1997, s 1K(6)).

39 For the tax year 2021/22, HMRC estimates that there were 68,800 individuals claiming non-dom taxpayer status in the UK, up from 68,000 in the tax year 2020/21; this is attributed to recovering arrivals numbers and a reduction in non-dom emigrations: HMRC, ‘Statistical Commentary on Non-domiciled Taxpayers in the UK’ (Official Statistics, 6 July 2023) < https://www.gov.uk/government/statistics/statistics-on-non-domiciled-taxpayers-in-the-uk/statistical-commentary-on-non-domiciled-taxpayers-in-the-uk> accessed on 2 February 2024. In 2020/21, the most recent year for which such data is available, the estimated total amount to be paid by taxpayers claiming the remittance basis (aggregating income tax, capital gains tax, national insurance contributions and the remittance basis charge) rose to £1.23 billion, indicating (in HMRC’s analysis) that the number of remittance basis users staying in the UK long enough to pay the remittance basis charge has been steadily increasing since the deemed domicile reforms in 2017.

40 The Italian non-subscription or ‘lump-sum’ regime for high-net-worth individuals was inserted as art 24-*bis* of the Italian Tax Code by Budgetary Law No 232/2016. For a detailed overview, see Francesco Oliveti, ‘New Italian Tax Regime for High Net-Worth Individuals: Italy as the New Attractive Destination’ (2017) 23(10) Trusts and Trustees 1022.

41 Malta, like the UK, provides for remittance-based taxation for resident non-domiciliaries: art 4(1)(g) of the Income Tax Act 1949 of Malta (as amended). See Office of the Commissioner for Revenue of Malta, ‘Guidance Note: The Remittance Basis of Taxation for Individuals Under the Income Tax Act’ (Office of the Commissioner for Revenue, 17 August 2022) <https://cfr.gov.mt/en/individuals/Pages/Tax-Residence.aspx> accessed 12 September 2023.

42 See eg John Ward and Clair Scott-McAteer, ‘The Remittance Basis of Taxation in Ireland’ (2012) 44(2) Accountancy Ireland 65.

43 Israel’s Income Tax Ordinance (amendment 168).

44 Portugal Income Tax Code, art 81, discussed in Tiago Marreiros Moreira, ‘Portugal Promotes Itself as Gateway to the World’ (2010) 21 International Tax Review 69.

45 To be sure, recent developments have demonstrated that there is also a flip side to such choices: the risk that one’s assets may not be as well protected as those of full members. See eg Foreign, Commonwealth & Development Office, ‘Press Release: UK Hits Key Russian Oligarchs with Sanctions Worth up to £10 Billion’ (UK Government, 14 April 2022) < www.gov.uk/government/news/uk-hits-key-russian-oligarchs-with-sanctions-worth-up-to-10bn> accessed 18 September 2023.

46 Wolfgang Schoen, ‘Taxation and Democracy’ (2018–19) 72 Tax L Rev 235.

47 Indeed, the availability of the option to exit and make a living elsewhere is key, even for those who choose not to exercise it, as it emphasises the choice one makes in not leaving. When one has the option to leave and still chooses to stay, their living within the community is more meaningful; for a more elaborate analysis, see Hanoch Dagan and Michael Heller, ‘The Liberal Commons’ (2001) 110(4) Yale LJ 549.

48 Charles Tiebout, ‘A Pure Theory of Local Expenditures’ (1956) 64(5) Journal of Political Economy 416. Tiebout’s model was conceived in the domestic inter-jurisdiction context as an alternative for determining the optimal level of production of public goods and services. For the applicability of the Tiebout model to international income taxation, see Dagan, *International Tax Policy* (n 22) 122–3.

49 For a review of these arguments, see Dagan, *International Tax Policy* (n 22) 120–41.

50 Discussing the responses to wealth tax particularly in the UK context, Advani and Tarrant observe: ‘On balance, the existing evidence suggests that migration responses to all forms of taxation are small relative to potential revenue, and there is little support for the view that the emigration of wealthy taxpayers poses a significant threat to progressive taxation.’ Income tax, however, seems a little different: ‘Personal income taxes are generally found to induce small although statistically significant responses among wealthy taxpayers, with the elasticity of the stock of wealthy taxpayers ranging from 0.03 to 2.109.’ See Arun Advani and Hannah Tarrant, ‘Behavioral Responses to a Wealth Tax’ (2021) 42 Fiscal Studies 509, 531–2. The OECD’s survey of the issue similarly suggests that income tax concessions tend to have a significant positive effect on migration, though the extent to which the concession is taken up appears to hinge on how ‘targeted’ the concession is: OECD, *Taxation and Employment* (n 20) 128–9, 139.

51 Dagan and Fisher (n 20).

52 Albert Hirschman, *Exit, Voice and Loyalty* (Harvard UP 1972).

53 One may think that a non-subscription option exacerbates the problem, but because non-subscribers fully exit the community, the question of horizontal equity is muted with respect to them.

54 Hirschman (n 52).

55 Chauvinist populism may be a pathological response to such a predicament. The more we see free agents who opt for the non-subscription option, the less viable is the notion that a state is organised as a stable political community which treats its constituents as civic members and is committed to treating them with equal concern and respect.

56 For an analysis suggesting no significant behavioural response to the deemed domicile reforms in the UK, see eg Arun Advani, David Burgherr and Andy Summers, ‘Reforming the Non-dom Regime: Revenue Estimates’ (2022) CAGE Policy Briefing 38.

57 Ruth Mason, ‘Citizenship Taxation’ (2016) 89 S Cal L Rev 169; Reuven Avi-Yonah, ‘Taxing Nomads: Reviving Citizenship-Based Taxation for the 21st Century’ in Tsilly Dagan and Ruth Mason (eds), *Taxing People: The Next 100 Years* (CUP forthcoming). When tax is imposed on a citizenship basis, renunciation of citizenship is necessary for the taxpayer to exclude themselves from the tax net; for an analysis of data on individuals who have renounced US citizenship suggesting that tax-motivated renunciations are uncommon (accounting for only 17,000 of four million renunciations in the dataset), see Paul Organ, ‘Citizenship and Taxes’ (2022) International Tax and Public Finance 1, 42.

58 See eg the migration patterns noted in Dumont and Lemaître (n 23) 1295 (tentatively concluding that OECD countries are the beneficiaries of the mobility of highly skilled workers) and the effectiveness of tax incentives to migration as documented by the OECD, *Taxation and Employment* (n 20); Flamant, Godar and Richard (n 20).

59 To clarify: the recent co-operative accord led by the OECD focused on corporate taxation, and made a concrete effort to allow states to tax large multinationals; it did not target individuals. And yet, a parallel effort (ie setting a minimum tax) can be imagined for individuals.

## Notes

I would like to thank Shai Agmon, Reuven Avi Yonah, Yariv Brauner, Emma Chamberlain, Hanoch Dagan, Mike Deveraux, David Enoch, Assaf Hamdani, Mindy Hertzfeld, Ruth Mason, Alice Pirlot, Linda Sugin and John Vella for their comments, and Louis Triggs for his outstanding research assistance. The research for this article was generously supported by an Oxford Law Faculty starting grant.

